# A scaffold-level genome assembly of a minute pirate bug, *Orius laevigatus* (Hemiptera: Anthocoridae), and a comparative analysis of insecticide resistance-related gene families with hemipteran crop pests

**DOI:** 10.1186/s12864-021-08249-y

**Published:** 2022-01-11

**Authors:** Emma Bailey, Linda Field, Christopher Rawlings, Rob King, Fady Mohareb, Keywan-Hassani Pak, David Hughes, Martin Williamson, Eric Ganko, Benjamin Buer, Ralf Nauen

**Affiliations:** 1 Department of Biointeractions and Crop Protection, Rothamsted Research, Harpenden, UK; 2 Department of Computational and Analytical Sciences, Rothamsted Research, Harpenden, UK; 3 The Bioinformatics Group, Cranfield Soil and Agrifood Institute, Cranfield University, Cranfield, UK; 4 Syngenta Biotechnology Inc, Research Triangle Park, NC USA; 5 Bayer AG, Crop Science Division, R&D, Monheim, Germany

**Keywords:** *Orius laevigatus*, Pirate bug, PacBio, Illumina, Whole genome sequencing, Beneficial predator, Insecticide resistance, Comparative genomics, Hemiptera, Crop pests

## Abstract

**Background:**

*Orius laevigatus*, a minute pirate bug, is a highly effective beneficial predator of crop pests including aphids, spider mites and thrips in integrated pest management (IPM) programmes. No genomic information is currently available for *O. laevigatus*, as is the case for the majority of beneficial predators which feed on crop pests. In contrast, genomic information for crop pests is far more readily available. The lack of publicly available genomes for beneficial predators to date has limited our ability to perform comparative analyses of genes encoding potential insecticide resistance mechanisms between crop pests and their predators. These mechanisms include several gene/protein families including cytochrome P450s (P450s), ATP binding cassette transporters (ABCs), glutathione S-transferases (GSTs), UDP-glucosyltransferases (UGTs) and carboxyl/cholinesterases (CCEs).

**Methods and findings:**

In this study, a high-quality scaffold level de novo genome assembly for *O. laevigatus* has been generated using a hybrid approach with PacBio long-read and Illumina short-read data. The final assembly achieved a scaffold N50 of 125,649 bp and a total genome size of 150.98 Mb. The genome assembly achieved a level of completeness of 93.6% using a set of 1658 core insect genes present as full-length genes. Genome annotation identified 15,102 protein-coding genes - 87% of which were assigned a putative function.

Comparative analyses revealed gene expansions of sigma class GSTs and CYP3 P450s. Conversely the UGT gene family showed limited expansion. Differences were seen in the distributions of resistance-associated gene families at the subfamily level between *O. laevigatus* and some of its targeted crop pests. A target site mutation in ryanodine receptors (I4790M, PxRyR) which has strong links to diamide resistance in crop pests and had previously only been identified in lepidopteran species was found to also be present in hemipteran species, including *O. laevigatus*.

**Conclusion and significance:**

This assembly is the first published genome for the Anthocoridae family and will serve as a useful resource for further research into target-site selectivity issues and potential resistance mechanisms in beneficial predators. Furthermore, the expansion of gene families often linked to insecticide resistance may be an indicator of the capacity of this predator to detoxify selective insecticides. These findings could be exploited by targeted pesticide screens and functional studies to increase effectiveness of IPM strategies, which aim to increase crop yields by sustainably, environmentally-friendly and effectively control pests without impacting beneficial predator populations.

**Supplementary Information:**

The online version contains supplementary material available at 10.1186/s12864-021-08249-y.

## Background

Loss of crops to insect pests can account for ~ 10% of potential yield, as a result of both direct feeding damage and the transfer of viral plant diseases [1]. Thus, to maximise crop yields and sustain food production for a growing world population, pests need to be controlled. At present this control relies mainly on the use of synthetic pesticides, many of which are non-selective and are therefore toxic to both their target pest species and to beneficial predators and parasitoids. As a result there may be a reduction in the predator populations to a level where they are no longer able to contribute natural pest control. This, along with the development of insecticide resistance in pests, can lead to pest populations surging, sometimes to even higher levels than pre-pesticide application [2–4]. Beneficial predators, such as those in the genus *Orius,* have proven to be especially effective in the biological control of crop pests [5]. As generalist predators, *Orius* species target a wide variety of pest species including aphids, beet armyworm, leafhoppers, mites, thrips and whiteflies, many of which are the world’s most damaging crop pests [6, 7]. Some *Orius* species are commercially available as biological control agents and are widely used for this purpose as part of integrated pest management (IPM) strategies, especially in covered crops [8–10].

Whole genome sequences of insects are helping us to understand many aspects of their biology and behaviour, and this can be applied to potential insecticide resistance mechanisms in pest insects and their natural enemies. However, only a few genomes of beneficial predator species have been published to date, including a phytoseiid mite, *Galendromus occidentalis* [11]; three parasitoid wasps, *Nasonia giraulti, Nasonia longicornis* and *Nasonia vitripennis* [12] and two lady beetles, *Harmonia axyridis* and *Coccinella septempunctata* [13]. To date there are no published genomes for species of the Hemiptera: Anthocoridae (i.e. minute pirate bug) family of predators. In contrast, a growing number of genomes of crop pests are available [14–26]. This larger number of pest genomes, relative to beneficial predator genomes could be in part because up until recently, the genomes of the pests themselves have appeared more useful in terms of developing targeted pesticides and investigating mechanisms of pesticide resistance. However, agriculture is now moving increasingly away from pesticide use – particularly with the Directive on Sustainable Use of Pesticides 2009/128/EC [27] - and towards IPM strategies, which includes the use of beneficial predators. Future studies of pesticide resistance mechanisms should therefore include beneficial predator genomes alongside pest genomes in order to help select targeted pesticides which do not harm beneficials and subsequently improve the efficacy of IPM strategies [28–32].

The aim of the work reported here was to develop a high-quality genome assembly for *O. laevigatus*, to serve as a resource for research into this species as well as the wider Anthocoridae family, which consists of 400–600 mostly predaceous minute pirate bug species - a potentially valuable source of biological control agents [33]. The *O. laevigatus* genome was then used for comparative analyses between beneficial predators and crop pests, focusing on genes encoding potential insecticide resistance mechanisms.

There are two main types of insecticide resistance mechanisms: increased expression of genes encoding protein families involved in metabolic resistance and point mutations in genes encoding insecticide target proteins [34]. Gene families involved in insecticide resistance in pest species are known to include cytochrome P450 monooxygenases (P450s), ATP binding cassette transporters (ABCs), glutathione S-transferases (GSTs), UDP-glucosyltransferases (UGTs) and carboxyl/cholinesterases (CCEs) [35–40]. Comparisons of the genes/proteins which may be involved in insecticide resistance in crop pests with the corresponding genes in beneficial insects, could aid the development of insecticides which target crop pests but have limited impact on beneficial predator populations. This could prove key to developing successful IPM strategies which exploit differences in the ability of predators and crop pests to tolerate pesticides. Improving the availability of beneficial predator genomes could also help the selection of beneficial predators with genes/mutations for inherent insecticide resistance before being released in the field for biological control [41].

The results presented here provide a comprehensive foundation for further study of potential insecticide resistance mechanisms in beneficial predators and how they compare to crop pests.

## Methods

### Sample preparation and sequencing

*Orius laevigatus* (commonly known as a minute pirate bug) were obtained from ‘Bioline AgroSciences’. CO_2_ was used for anaesthesia to allow the insects to be sorted from the substrate. Both adults and nymphs were then flash frozen with liquid N_2_ and stored at − 80 °C. The whole process was done within 48 h of arrival.

~ 1000 individuals were pooled for genomic DNA/RNA extractions, which were carried out in-house at Rothamsted Research. The commercial DNAzol reagent was used for the DNA extractions, and the Bioline Isolate II RNA Mini Kit was used for the RNA extractions. The DNA and RNA were sent for library preparation and sequencing by Genewiz (New Jersey, US).

The genome assembly was developed using a hybrid assembly strategy with both Illumina short reads and Pacific Biosciences (PacBio) long reads.

Short reads were sequenced using 2 mg of DNA and a library with an insert size of 200 bp. Sequencing was done using Illumina HiSeq 4000 with a 2x150bp paired-end configuration. 413,143,574 reads were obtained with a total length of 123 Gb (820x). Raw reads are available under SRA accession: ERR6994870. K-mer counting of the raw Illumina DNA data was done using Jellyfish 2.2.6 [42]. Canonical (−C) 21-mers (−m 21) were counted and a histogram of k-mer frequencies outputted. GenomeScope 2.0 [43] was used to process this histogram with ‘ploidy’ set at 2 and ‘maximum k-mer coverage cut-off’ set at 10,000.

To obtain long read PacBio data, 3.7 mg of DNA first underwent blue pippin size selection (> = 10 kb) to remove low molecular weight DNA. < 500 ng of DNA remained after size selection, and so a low input protocol was used for library construction with an insert size of 20 kb. Sequencing was done using the PacBio Sequel I platform and 537,651 reads were obtained with a total length of 6Gb (44x) and an N50 of 11,287 bp. Raw reads are available under SRA accession: ERR6941611.

Transcriptome sequencing used 10 mg of RNA and a library construction with an insert size of 150 bp and PolyA selection for rRNA removal. Sequencing was done using Illumina HiSeq 4000 with a 2x150bp paired-end configuration. 413,137,378 reads were obtained. Raw reads are available under SRA accession: ERR7012629.

FastQC v.0.11.8 [44]. was used for quality checks on the raw Illumina HiSeq DNA and RNA sequence data. Adapters were trimmed, low-quality bases (below a score of 3) were removed from the start and end of reads and any reads with a length less than 36 bases were also removed. Trimmomatic v.0.38 [45]. was used for these trimming steps. Quality trimming of reads using Trimmomatic resulted in a 0.2% loss of reads for whole genome sequencing and a 5% loss of reads for transcriptome sequencing (Table 1).

**Table 1:** Number of paired-end Illumina HiSeq DNA sequences present before and after trimming

	Illumina DNA Reads	Illumina RNA Reads
Total sequences before trimming	413,143,574	413,137,378
Total sequences after trimming	412,474,208	389,150,727
Sequences lost	669,366	23,986,651

### Genome quality assessment

Basic metrics from the genome assembly were calculated using a script developed for the ‘Assemblathon’ [46]. These metrics include scaffold/contig N50, longest and shortest scaffold length, number of scaffolds exceeding a range of lengths and number of gaps/N’s in the assembly.

The completeness of the genome assembly and annotation for *Orius laevigatus* was assessed using the Benchmarking Universal Single-Copy Orthologs (BUSCO) [47] of the insect gene set (insecta odb9). ‘Genome’ mode was used to assess the assembly, and ‘protein’ mode to assess the annotation. ‘Fly’ was used as the training species for Augustus gene prediction. BUSCO assessments were then run with default parameters.

### De novo genome assembly

**Fig. 1:**
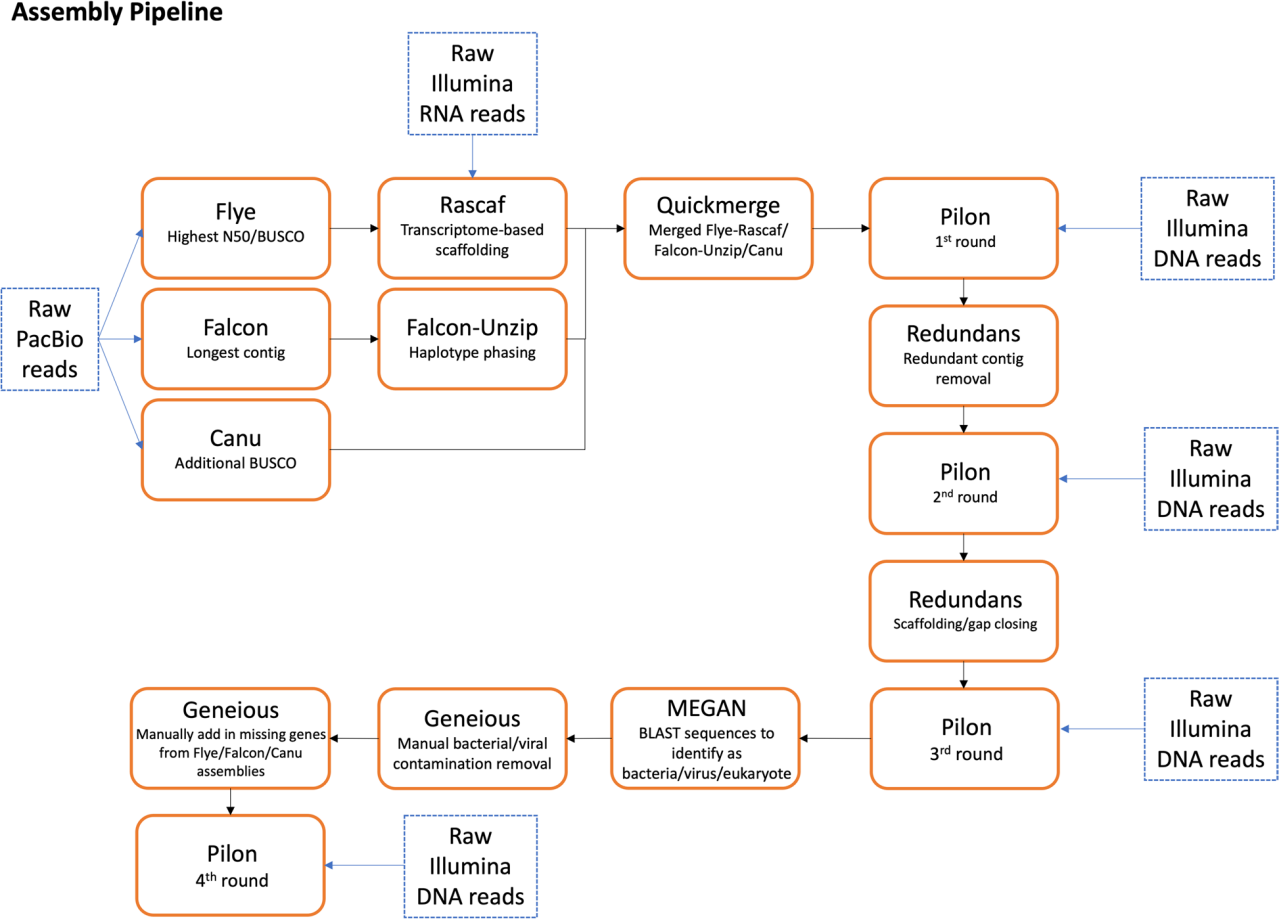
The assembly pipeline for the *Orius laevigatus* genome

The overall assembly pipeline is shown in Fig. 1. The raw PacBio long reads were assembled into contigs with the Flye v2.5. de novo assembler [48, 49]. Rascaf was then used to improve the Flye genome assembly with RNA-seq data [50]. Contigs were also produced with the raw PacBio long reads using Canu v1.8 [51] as well as with FALCON v1.3.0 and FALCON-Unzip, which is recommended for heterozygous/outbred organisms with diploid or higher ploidy (and also includes phased-polishing with Arrow) [52, 53].

QuickMerge v0.3 [54] was used to merge the assemblies, with Flye as the reference assembly. BUSCO outputs were compared between the merged assembly and the standalone assemblies to identify genes which had been lost during the merging process. Full-length contigs containing these missing genes were extracted from the standalone assemblies and added to the merged assembly, based on the assumption that these contigs would also contain other missed genes (i.e. those not included in BUSCO’s list of 1658 core insect genes). Multiple rounds of Pilon error polishing [55] were performed, using the Illumina short read data, until no further improvement in BUSCO score was seen.

Redundans [56] was used for scaffolding and redundant contig removal. Redundans is geared towards highly heterozygous genomes. Some redundant regions had to be removed manually, as Redundans does not detect redundancy when only part of the contig is duplicated. The nucmer tool from the MUMmer4 package [57] was used to detect these redundant regions through a whole genome self-alignment.

A BLAST search against the NCBI Reference Sequence (Refseq) database release 93 [58], was performed using the Tera-BLAST algorithm on a TimeLogic DeCypher system (Active Motif Inc., Carlsbad, CA). The results were processed with Megan [59] to identify any bacterial or viral sequences which were then removed manually in Geneious v10.2.6.

The mitochondrial genome sequence was identified and extracted by running a BLAST search of the *O. laevigatus* genome against the *Orius sauteri* mitochondrial genome which is publicly available at NCBI, GenBank accession No. KJ671626 [60].

### Genome annotation

Gene prediction was performed using the MAKER v2.31.8 pipeline [61] through the incorporation of both transcriptome evidence and ab initio gene prediction as well as a custom repeat library (see below). MAKER was run using Augustus v3.3.1 [62], GeneMark-ES v4.32 [63] and FGeneSH v8.0.0 [64] as well as EVidenceModeler v1.1.1 [65] with default masking options.

A de novo species specific repeat library was constructed using RepeatModeler v1.0.7 [66] to identify repeat models. These models were searched against the GenBank non-redundant (*nr*) protein database for Arthropoda (e value < 10^− 3^) using Blastx to remove any potential protein-coding genes. This was combined with transposon data to create a custom library. Transposons were identified from the transcriptome assembly by running HMMER: hmmscan [67] against the Pfam database [68] and filtering the resultant Pfam descriptions for those containing “transposon”. A search for transposons was also done on transcripts produced from MAKER and these transposons were then added to the custom repeat library which was used for a second round of MAKER. RepeatMasker v4.0.7 [69] was used to mask repeats in the genome assembly using these repeat libraries, as well as to estimate the abundances of all predicted repeats.

RNA-seq reads were mapped to the genome with HISAT2 v2.0.5 [70] for assembly with StringTie v1.0.1 [71]. A de novo assembly was also done using Trinity v2.5.1 [72]. The best transcripts (classified by reasonable transcript size and homology to other species) were selected from the Trinity and StringTie assemblies using Evigene v19.jan01 [73].

Evidence from assembled transcripts was transferred to the genome assembly via MAKER. The output from this was then used to produce a high confidence level gene model training set - overlapping and redundant gene models were removed. Augustus and GeneMark were trained using this training set prior to being used for ab initio gene predictions. FGeneSH was run based on the *Drosophila melanogaster* genome.

The best transcripts from both the ab initio gene prediction annotation and the transcriptome-based annotation were selected using Evigene (classified by reasonable protein size and homology to other species) and combined to create the final annotation.

*Orius laevigatus* protein sequences were aligned using Blastp against the non-redundant (*nr*) NCBI protein database for Arthropoda. InterProscan searches were run against several databases (CDD, HAMAP, HMMPAnther, HMMPfam, HMMPIR, FPrintScan, BlastProDom, ProfileScan, HMMTigr) for functional annotation. BLAST2GO [74] was used to assign gene ontology (GO annotations). Infernal v1.1.2 [75] was used to predict and annotate non-coding RNAs.

The mitochondrial genome was annotated using MITOS2 [76] with reference database ‘RefSeq 81 Metazoa’ and genetic code ‘5 Invertebrate’.

### Comparative genomics and phylogenetic analysis

To produce the species tree, orthogroup gene trees were produced using Orthofinder [77] and the tree was inferred from these using the STAG method [78].

In order to identify genes potentially involved in insecticide resistance, the PFAM domains assigned to gene models during annotation (as described in the ‘Genome Annotation’ methods section) were used as follows: CCEs (PF00135/IPR002018), GSTs (IPR004045/PF02798), (IPR004046/PF00043), P450s (IPR001128/PF00067), ABCs (IPR003439/PF00005) and UGTs (IPR002213/PF00201). Proteins from UniProt for the classes of interest, from hemipteran species, were used for BLAST queries against *O. laevigatus* to identify any missed genes and to assist with subfamily assignment within these classes. Subfamily assignment for *O. laevigatus* gene families was finalised using phylogenetic trees produced using MAFFT alignments [79, 80] and RaxML v8.2.11 [81]. The GAMMA LG protein model [82] was used and a bootstrap consensus tree was inferred from 100 replicates.

Manual checks and curation were performed for genes potentially involved in insecticide resistance. Increased copy numbers of these genes often led to adjacent tandem duplications being incorrectly annotated as one gene model, therefore curation was important to prevent incorrect gene numbers being reported in later analyses. The exon/intron boundaries and start/stop codons of the genes were confirmed through visualization in IGV [83] of RNAseq data mapped to the genome using HISAT2 v2.0.5 [70] and the gene models were edited in Geneious where necessary.

The P450s were classified and named by Dr. David Nelson [84]. The UGTs were classified and named by Dr. Michael Court [85]. Nomenclature of P450s and UGTs is based on the evolutionary relationships of the sequences. P450 and UGT sequences were BLAST searched against named insect sequences and were assigned to known families if they were > 40% (for P450 families) or > 45% (for UGT families) identical. Other sequences were assigned to new families based on their clustering on trees and their percent identity to each other.

## Results and discussion

### Sequencing

In order to produce enough DNA and RNA for sequencing, ~ 1000 individuals of *O. laevigatus* were required. Because they were obtained commercially, the level of inbreeding of the culture was not known. However, all individuals were obtained from a single colony within the rearing facility. A high heterozygosity level was therefore a possibility and this was kept in mind when making decisions during the assembly process.

### Genome metrics evaluation based on raw reads

The raw read k-mer analysis with GenomeScope 2.0 estimated a haploid genome size of ~ 141 Mb (Table 2), in line with the final assembly size of 151 Mb. A genome size estimate using methods such as flow cytometry would have provided a more accurate estimate, however, such data was not available for the *Orius* genus. This could be considered a limitation to the study, as 141 Mb was provided as a genome size estimate to Canu, Flye and FALCON-Unzip which may have affected the outputted assemblies. Genome repeat length was 20 Mb, 16.5% of the total estimated genome size.

**Table 2:** Genome characteristics obtained from GenomeScope v2.0 Using a k-mer length of 21 and a maximum k-mer coverage of 10,000

	Minimum	Maximum
Heterozygosity, %	1.197	1.297
Genome Haploid Length (Mb)	140.7	142.2
Genome Repeat Length (Mb)	20.2	20.5
Genome Unique Length (Mb)	120.4	121.8
Read Error Rate, %	0.86	0.86

**Fig. 2:**
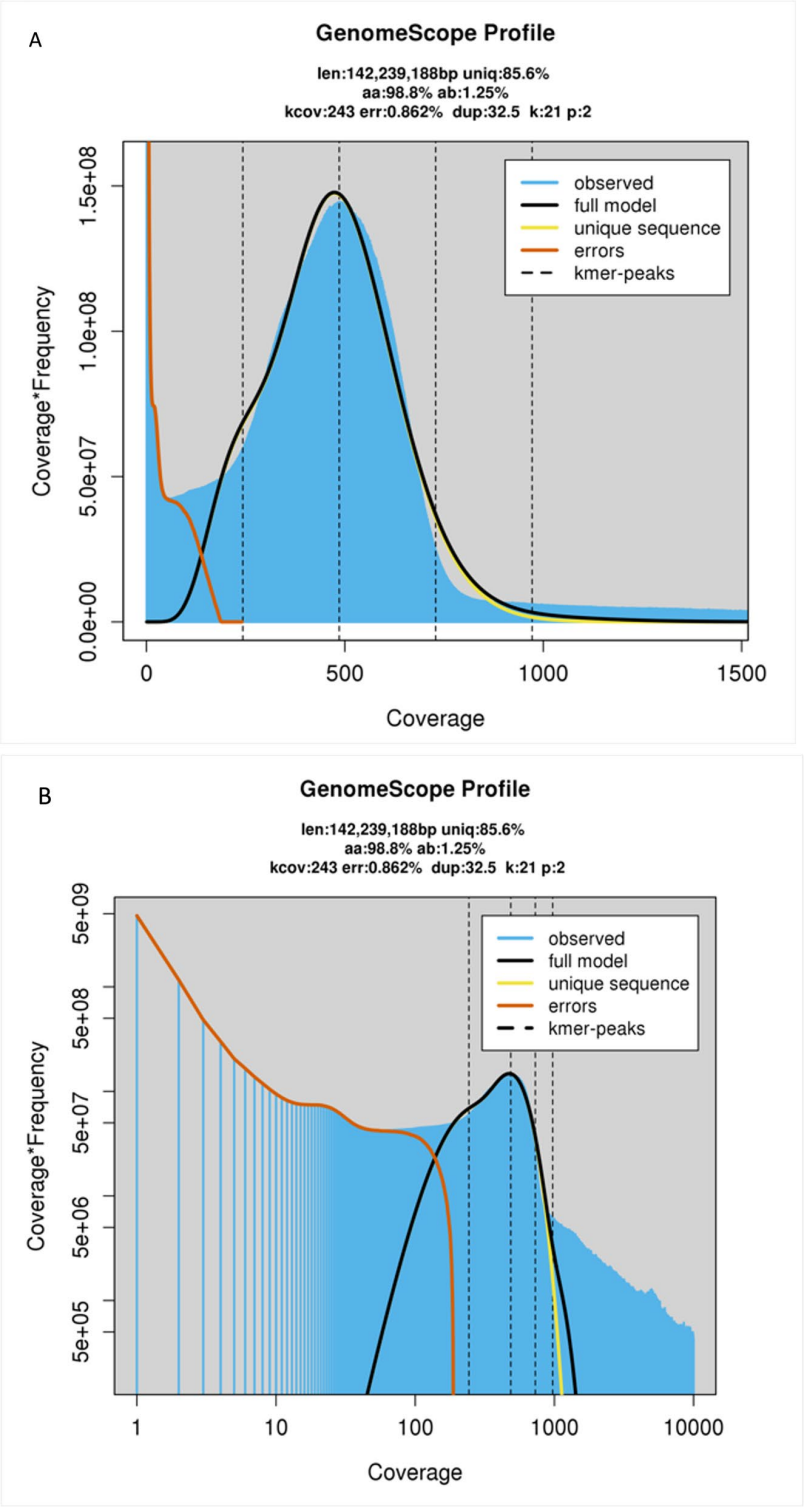
GenomeScope v2.0 profile plots of **A**: a transformed linear plot of k-mer frequency; and **B**: a transformed log plot of k-mer coverage at a k-mer length of 21 and a maximum k-mer coverage of 10,000

The heterozygosity rate ranged from 1.20 to 1.30%. This alongside the small ‘shoulder’ to the left of the main ‘full-model’ peak (Fig. 2), indicates a fairly high level of heterozygosity, which was taken into consideration in the assembly strategy.

### Assembly

Flye, FALCON and Canu were used to produce 3 separate genome assemblies. The statistics for these assemblies, as well as for subsequent versions of the assembly outlined in this section are shown in Additional file 2. Rascaf improved the contiguity of the Flye assembly through alignment of the RNA-seq data to the genome, likely because it is less affected by the use of multiple individuals versus genome assembly tools which include non-conserved sequences from a population of individuals. FALCON-Unzip improved the FALCON assembly contiguity with a 4.5-fold decrease in the total number of scaffolds (although this coincided with a ~ 9% loss of complete gene models found using BUSCO and suggests that FALCON-Unzip may have been too stringent for this genome - perhaps because it was designed with plant and fungal genomes in mind [52, 53]).

Flye (both with and without Rascaf) had the best assembly statistics in terms of scaffold N50 and BUSCO score. However, FALCON-Unzip achieved the largest ‘longest scaffold’ of the three assemblers.

Quickmerge was used to merge the FALCON-Unzip assembly, Rascaf improved Flye assembly and the Canu assembly. The resultant merged assembly had better continuity than any of the stand-alone assemblies, however, the BUSCO completeness was slightly worse than the standalone Flye assembly (and worsened with the second round of Quickmerge). This was likely due to mis-assemblies in the component assemblies causing alignment issues, which resulted in sections of the misassembled contigs being discarded.

Pilon was used for error polishing and improved the BUSCO completeness score. Redundans (redundancy removal and scaffolding/gap-closing) improved the scaffold N50 and removed redundant scaffolds.

A comparison of the gene models (core insect genes from the insecta odb9 BUSCO gene set) found in the original Flye / FALCON-Unzip / Canu assemblies versus the merged assembly showed that some of the gene models were found in at least one of the original assemblies, but were missing in the merged assembly. Of the 154 missing or fragmented genes in the merged assembly (out of a total 1658 core insect genes), 5 were found in the FALCON-Unzip assembly, 5 in the Flye assembly and 46 in the Canu assembly. Manual editing to bring the full-length contigs containing these missing genes into the merged assembly took the BUSCO completeness score up by 5%. A final round of Pilon improved this score by an additional 0.5% (further rounds of Pilon did not improve the score).

This brought the final assembly statistics to 93.6% BUSCO (insecta) complete, scaffold N50: 125,649 bp, the longest scaffold: 2,051,674 bp and 89.4% of scaffolds > 10 k in length (Table 3). The final assembly is available under GenBank accession: GCA_018703685.1. Transcriptome sequences are available under accessions: HBWI01000001-HBWI01209903.

**Table 3:** Final assembly statistics for the *O. laevigatus* genome

**Number of scaffolds**	2050
**Total size of scaffolds**	150,957,203 bp
**Longest scaffold**	2,051,674 bp
**Shortest scaffold**	1007 bp
**Number of scaffolds > 1 K nt**	2050 (100.0%)
**Number of scaffolds > 10 K nt**	1832 (89.4%)
**Number of scaffolds > 100 K nt**	386 (18.8%)
**Number of scaffolds > 1 M nt**	4 (0.2%)
**Number of scaffolds > 10 M nt**	0 (0.0%)
**N50 scaffold length**	125,649 bp
**Number of N’s**	21, 965 *
**Number of gaps**	187 *

*(1 gap was 17,239 N’s, and another gap was 1243 N’s. All other gaps were < 100 N’s)

### Annotation

Gene prediction with MAKER identified 15,102 protein-coding genes with the encoded proteins having a mean length of 464 amino acids. Of these, 12,949 (86%) had a match to NCBI’s non-redundant (*nr*) database and 11,616 (77%) contained InterPro motifs, domains or signatures. In total, 13,112 (87%) were annotated with either blastp or InterPro and 10,192 were annotated with a GO ID. More information on the InterPro member database annotations is given in additional file 1. The longest protein found was an ‘egf-like protein’ at 14,628 amino acids. The resultant gene set was 84.5% BUSCO (insecta) complete.

From the Infernal tool inference of RNA alignments, a total of 791 non-coding RNA elements and 269 *cis*-regulatory elements were found in the genome (Table 4).

**Table 4:** Number of ncRNAs predicted in the *Orius laevigatus* genome

ncRNA element	Number of elements
tRNA	503
rRNA	182
snRNA	53
miRNA	41
srpRNA	6
snoRNA	3
lncRNA	3

### Repeat annotation

Transposable and repetitive elements made up 27.07% of the assembled *O. laevigatus* genome (Table 5) and the majority of these (20.4%) were unclassified repeats. This is close to the reported repeat content of other hemipteran species, for example: *Cimex lectularius -* 31.63% [86] and *Acyrthosiphon pisum* - 38% [15], an exception is *Rhodnius prolixus* which has an unusually low repeat content of 5.6% [87].

**Table 5:** Summary of transposable and repetitive elements in the *Orius laevigatus* genome

	Number of Elements	Length Occupied	Percentage of Sequence
**SINES**	705	59,683 bp	0.04%
**LINES**	3309	1,556,653 bp	1.03%
LINE1	0	0 bp	0.00%
LINE2	496	257,681 bp	0.17%
L3/CR1	2310	890,133 bp	0.59%
**LTR elements**	959	501,171 bp	0.33%
**DNA elements**	5490	1,715,984 bp	1.14%
hAT-Charlie	784	222,164 bp	0.15%
TcMar-Tigger	99	41,650 bp	0.03%
**Unclassified**	105,531	30,830,578 bp	20.42%
**Total interspersed repeats**	**NA**	**34,664,069 bp**	**22.96%**
**Small RNA**	127	35,035 bp	0.02%
**Satellites**	4867	3,456,707 bp	2.29%
**Simple repeats**	30,022	2,273,603 bp	1.51%
**Low complexity**	7742	444,236 bp	0.29%
**Total:**	**NA**	**42,285,278 bp**	27.07%

### Mitochondrial genome

**Fig. 3:**
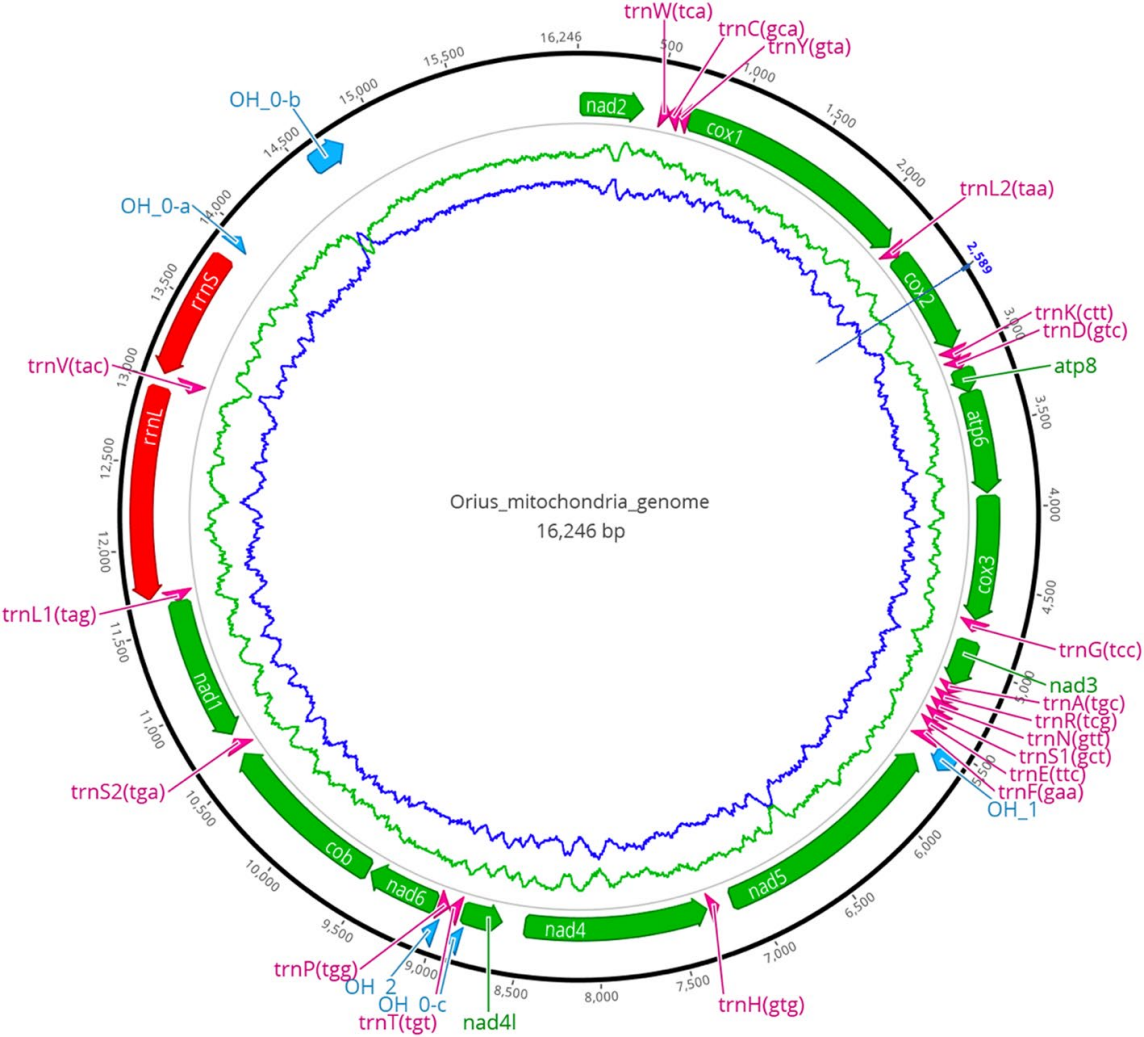
The mitochondrial genome for *Orius laevigatus,* visualised using Geneious and annotation track obtained using MITOS2. The innermost graphs represent AT content shown in green, and GC content shown in blue

A circularized mitochondrial genome of 16,246 bp, assembled and annotated using MITOS2, consisted of 13 protein coding genes, 19 tRNA genes, 2 rRNA genes and an A + T rich region with a length of 1460 bp and an A + T content of 72.7% (Fig. 3). This closely matches the *Orius sauteri* mitochondrial genome, which is also 16,246 bp and has 13 protein-coding genes, 22 tRNA genes, 2rRNA genes and an A + T rich region of 1758 bp and an A + T content of 73.5% [60].

### Phylogeny

OrthoFinder assigned 318,985 genes (88.8% of total) to 27,481 orthogroups. There were 1621 orthogroups with all species present and 45 of these consisted entirely of single-copy genes.

**Fig. 4:**
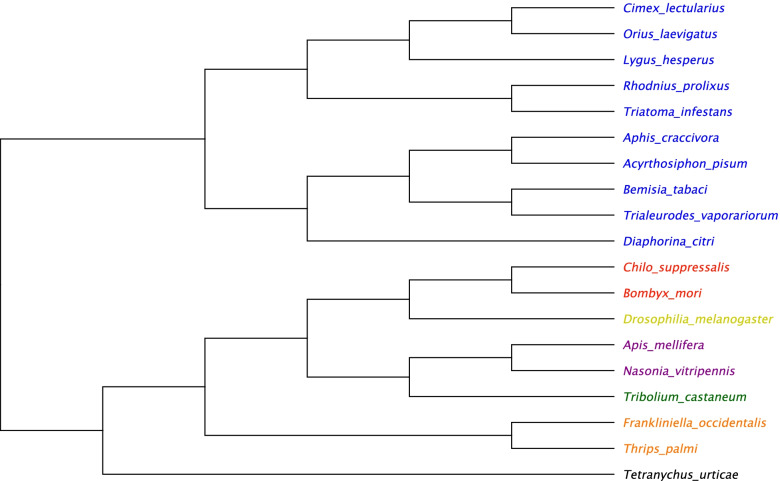
Phylogeny and divergence of Insecta. Nodes are coloured by order, blue = Hemiptera, red = Lepidoptera, yellow = Diptera, purple = Hymenoptera, green = Coleoptera, orange = Thysanoptera, black = Chelicerata. Produced using the STAG tree inference method and full proteomes of the following species: *C. lectularius:* PRJNA167477, *L. hesperus*: PRJNA284294, *R. prolixus:* PRJNA13648, *T. infestans*: PRJNA589079, *A. craccivora:* PRJNA558689, *A. pisum*: PRJNA13657, *B. tabaci*: PRJNA312470, *T. vaporariorum*: PRJNA553773, *D. citri*: PRJNA2944, *C. suppressalis*: PRJNA506136, *B. mori:* PRJNA205630, *D. melanogaster*: PRJNA13812, *A. mellifera*: PRJNA471592, *N. vitripennis*: PRJNA575073, *T. castaneum*: PRJNA12540, *F. occidentalis*: PRJNA203209, *T. palmi*: PRJNA607431, *T. urticae:* PRJNA315122

Phylogenetic analysis correctly clustered *O. laevigatus* within the hemipteran clade (Fig. 4) and identified *Cimex lectularius* as its closest relative.

### Comparative genomics

#### ABC transporters

ATP-binding cassette transporters (ABCs), the largest known group of active transporters, can eliminate xenobiotic compounds - such as secondary metabolites produced by plants or insecticides - through translocation [36]. These transporters are subdivided into eight subfamilies: ABCA-H. ABCB, ABCC and ABCG are the subfamilies most associated with resistance to a variety of insecticides including pyrethroids, carbamates, organophosphates and neonicotinoids [88]. Forty-one of the 64 transporters in *O. laevigatus* belong to these 3 class-specific expansions (Table 6) which could confer resistance to insecticides (a phylogenetic tree showing relationships of ABC transporters in *O. laevigatus* is included in Additional file 3, full sequences are included in Additional file 4).

**Table 6:** Numbers of ABC transporter genes annotated in *Orius laevigatus* (this study), *Cimex lectularius* [86], *Lygus hesperus* [89], *Frankliniella occidentalis* [90], *Thrips palmi* [91], *Aphis gossypii* [92], *Trialeurodes vaporariorum* [93], *Diuraphis noxia* and *Bemisia tabaci* [94]

	*O. laevigatus* + close relatives			Crop pests					
	***O. laevigatus***	***C. lectularius***	***L. hesperus***	***F. occidentalis***	***T. palmi***	***D. noxia***	***A. gossypii***	***T. vaporariorum***	***B. tabaci***
**ABCA**	11	6	11	3	3	3	4	3	8
**ABCB**	9	7	6	5	4	6	5	9	3
**ABCC**	9	6	12	19	12	24	25	7	6
**ABCD**	1	2	2	2	2	3	2	4	2
**ABCE**	1	1	1	1	2	1	1	1	1
**ABCF**	5	4	3	3	3	3	4	3	3
**ABCG**	23	23	19	22	16	26	30	9	23
**ABCH**	2	2	11	13	7	11	0	9	9
**Total**	**64**	**51**	**65**	**70**	**49**	**77**	**71**	**45**	**55**

Table 6 shows a comparison of numbers of ABC transporter genes found in the current study with those reported for some pest species. The gene family expansions were generally seen in the ABCC and ABCG classes for all hemipteran species and slightly larger expansions were seen in some crop pests compared to *O. laevigatus* for the ABCC class, however, the expansions were of very similar size for both crop pests and *O. laevigatus* in the ABCG class. Overall, the total numbers of ABC transporter genes were similar across all the hemipteran species compared.

#### Glutathione S-Transferases

The glutathione S-transferases (GSTs) protein family is large and functionally diverse, and is known to confer resistance to all main insecticide classes. GST-mediated detoxification of insecticides takes place via several different mechanisms, including protecting against oxidative stress, binding and sequestration of the insecticide, and by catalysing the conjugation of glutathione to the insecticide to reduce their toxicity [37].

**Fig. 5:**
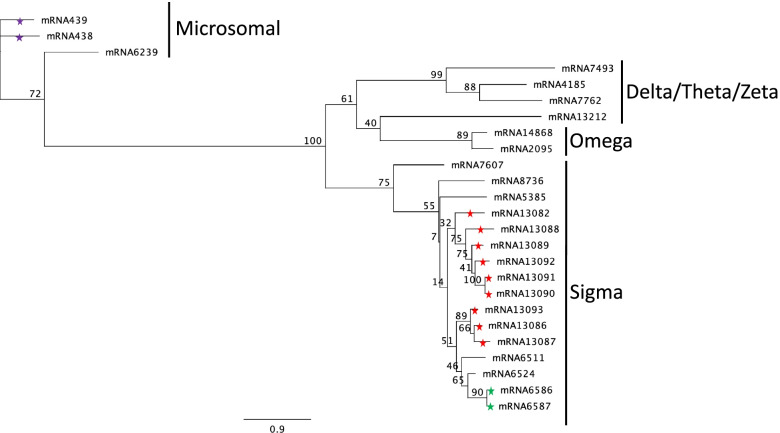
Phylogenetic tree of the *Orius laevigatus* glutathione S-transferases. Amino acid sequences were aligned using MAFFT and analysed using RAxML (the GAMMA LG protein model was used). The bootstrap consensus tree was inferred from 100 replicates. Coloured stars on branches indicate tandem duplications, with each colour representing a different scaffold/set of tandem duplications in the *O. laevigatus* assembly

The number of GST genes in *O. laevigatus* was fairly similar to other hemipteran close relatives, with the exception of the sigma class, which was notably larger (Table 7, full sequences included in Additional file 4). Of the 16 genes in the sigma class, 9 genes (mRNA13082 and mRNA13086–13,093) were adjacent on the same scaffold, indicating a lineage specific expansion (Fig. 5). Expansions in this class have been reported in several hemipteran species including *Triatoma infestans, Myzus persicae, Halyomorpha halys* and *Murgantia histrionica* [23, 24, 95, 96]. The sigma class has been found to play an important role in detoxification of organophosphorus insecticides in hemipteran species [97], therefore this expansion could potentially confer some tolerance to organophosphates in *O. laevigatus*. The delta and epsilon classes of GSTs are linked to insecticide resistance to pyrethroids [98, 99]. The delta class is much larger in several crop pests compared to *O. laevigatus* and its close relatives which suggests these crop pests could exhibit a higher level of delta class GST-mediated pyrethroid resistance. The epsilon class has previously been thought to be specific to Holometabola [100], and whilst *Trialeurodes vaporariorum* has a single member, the epsilon class is absent from all other Hemiptera species, suggesting potential epsilon class GST-mediated pyrethroid resistance is most likely absent in *O. laevigatus* and its close relatives, as well as most Hemiptera crop pests.

**Table 7:** Numbers of glutathione S-transferase genes annotated in *Orius laevigatus* (this study), *Cimex lectularius* [101], *Rhodnius prolixus, Triatoma Infestans* [96], *Thrips palmi* [91], *Myzus persicae, Acyrthosiphon pisum, Trialeurodes vaporariorum, Bemisia tabaci, Halyomorpha halys* [102] and *Murgantia histrionica* [24]

	*O. laevigatus* + close relatives				Crop pests						
	***O. laevigatus***	* C. lectularius	***R. prolixus***	***T. infestans***	***T. palmi***	***M. persicae***	***A. pisum***	***T. vaporariorum***	***B. tabaci***	***H. halys***	***M. histrionica***
**Delta**	1	1	1	1	14	3	11	9	14	2	4
**Epsilon**	0	0	0	0	0	0	0	1	0	0	0
**Omega**	2	1	1	0	1	1	1	0	1	3	0
**Sigma**	16	5	7	9	6	12	5	3	6	19	25
**Theta**	1	2	3	2	1	1	2	0	0	3	2
**Zeta**	1	1	1	0	2	0	0	2	2	1	0
**Microsomal**	3	0	1	2	1	2	2	3	2	5	3
**Total**	**24**	**10**	**14**	**14**	**25**	**19**	**21**	**18**	**25**	**33**	**34**

* *C. lectularius* numbers may be an underestimate as sequencing coverage was low for this study

#### Carboxyl/cholinesterases

Many carboxyl/cholinesterases (CCEs) are linked to detoxification of organophosphorus, carbamate and pyrethroid insecticides and acetylcholinesterase (AChE) is the target for organophosphate and carbamate insecticides, with amino acid substitutions being linked to resistance [39]. Thirty-two members of the CCE superfamily, including 1 AChE gene, were found in the *O. laevigatus* genome (Table 8, full sequences included in Additional file 4) which is a similar number to that reported for *Cimex lectularius*, which had 30 CCE genes and 2 AChE genes [86].

**Table 8:** Numbers of Carboxyl/cholinesterases annotated in Orius laevigatus (this study), *Cimex lectularius* [101], *Rhodnius prolixus* [103], *Triatoma infestans* [96], *Frankliniella occidentalis* [90], *Myzus persicae* [95], *Acyrthosiphon pisum, Bemisia tabaci* [104] and *Trialeurodes vaporariorum* [105] and their distribution across classes and clades

		*O. laevigatus* + close relatives				Crop pests				
		***O. laevigatus***	***C. lectularius***	***R. prolixus***	***T. infestans***	***F. occidentalis***	***M. persicae***	***A. pisum***	***T. vaporariorum***	***B. tabaci***
**Dietary class**		0	0*	22 (0)***	0	28	5	5	12	6
**Hormone/semiochemical processing class**		16	20*	9 (31)***	18	7	12	16	6	19
**Neuro- developmental class**	Glutactins	1	0*	2	0	2	0	0	1	1
	AChE	2	1*	2	1	2	3	2	2	4
	uncharacterised	1	1*	2	0	2	1	1	1	1
	gliotactin	3	0*	1	0	1	1	1	1	1
	neuroligin	8	0*	4	0	7	0	3	3	10
	neurotactin	1	0*	1	0	1	0	0	1	0
	Subtotal	16	2*	12	1	15	5	7	9	17
**Total**		**32**	22* (30)**	**43**	**19**	**50**	**22**	**28**	**27**	**42**

* *C. lectularius* numbers may be an underestimate as sequencing coverage was low for this study, clade assignment was also uncertain as a result

** A more recent study [86] found 30 CCE genes in *C. lectularius*, and is more likely to be a true representation, but they had not been assigned into classes/clades

*** Numbers in brackets represent the possible true numbers of *R. prolixus* CCEs, based on a potential misassignment of 22 genes to the dietary class instead of the hormone/semiochemical processing class

The dietary class of CCEs is involved in insecticide and xenobiotic detoxification [106]. *O. laevigatus* has no genes within this class, in line with *T. infestans* and *C. lectularius,* whereas the crop pest species (i.e. thrips, aphids and whiteflies) all have at least 5 members in this class (Table 8). *R. prolixus* has 22 genes which have been classed as dietary; however this assignment was based heavily on a species-specific expansion which is characteristic of the dietary class. The real number of genes in the dietary class for *R. prolixus* may be 0, since this clade of 22 genes clusters with the hormone/semiochemical class in both the *R. prolixus* study [103] and this study (Additional file 5). A lack of dietary esterases in *R. prolixus* would make sense, as *R. prolixus, C. lectularius* and *T. infestans* are all blood-sucking insects and do not require dietary esterases to process the secondary metabolites found in plants. This could also explain why *O. laevigatus,* a beneficial predator of crop pests in both nymph and adult life stages, does not require dietary esterases.

The dietary class is involved in pyrethroid resistance [107]; however, *T. infestans* exhibits pyrethroid esterase activity despite having no dietary esterases [108]. *O. laevigatus* has also shown the ability to develop pyrethroid resistance - although the exact mechanism of this resistance is not yet known [109]. The hormone and semiochemical processing class is also involved in insecticide metabolism, due to the presence of β-esterases [110, 111]. There may be some redundancy in genes potentially involved in insecticide detoxification from the dietary and hormone/semiochemical processing classes. This might explain why only one of these classes shows an increased number of genes for each of these hemipteran species (Table 8), as having increased numbers of both classes would be redundant, whilst very low numbers of both classes would be detrimental. The lack of the dietary class may therefore not impact the xenobiotic resistance abilities of *O. laevigatus,* as it has 16 genes within the hormone/semiochemical processing class.

The remaining CCEs in *O. laevigatus* belong to the neurodevelopmental class and include the neuroligins, gliotactins, glutactins and neurotactins, which are non-catalytic due to the lack of a critical serine residue. Acetylcholinesterase is the only protein in this class which has been linked to organophosphate resistance [112, 113].

#### UGTs

UDP-glucosyltransferases (UGTs) are detoxification enzymes speculated to be involved in insecticide metabolism. Although the exact mechanisms of UGT-mediated resistance have not yet been identified, their upregulation has been shown in resistant strains of *P. xylostella* [35] and they have been linked to diamide resistance in *Chilo suppressalis* [114], neonicotinoid resistance in *Diaphorina citri* [115] and they also contribute to insecticide detoxification via the elimination of oxidative stress in *Apis cerana* [116].

The number of UGT genes in *O. laevigatus* was much lower than for other hemipteran species (Table 9). The UGTs were submitted to Dr. Michael Court for naming and are included in Additional file 4. Numbers of UGTs have been reported to be lower in non-phytophagous insects [91], which could explain the low numbers seen in *O. laevigatus* and *R. prolixus* compared to crop pests. This suggests that UGT-mediated detoxification may be lower in *O. laevigatus* than in crop pests.

**Table 9:** Numbers of UDP glucuronosyltransferase genes found in *O. laevigatus* (this study), *Rhodnius prolixus, Tetranychus urticae, Nilaparvata lugens, Acyrthosiphon pisum, Bemisia tabaci* [19], *Myzus persicae* [117] and *Trialeurodes vaporariorum* [118]

	*O. laevigatus* + close relatives		Crop pests					
	***O. laevigatus***	***R. prolixus***	***T. urticae***	***N. lugens***	***M. persicae***	***A. pisum***	***T. vaporariorum***	***B. tabaci***
**Total**	10	16	81	20	101	72	55	76

#### Cytochrome P450s

Cytochrome P450s are a diverse superfamily capable of metabolizing a huge variety of endogenous and exogenous substrates. In insects they are associated with growth and development, metabolism of pesticides and plant toxins as well as the production and metabolism of insect hormones and pheromones. P450s are associated with resistance to insecticides from a variety of classes, including pyrethroids, carbamates and neonicotinoids. They are also linked to the activation of organophosphates and other pro-insecticides (a form of insecticide which is metabolized into an active form inside the host) [38]. Upregulation of P450s in insects has been shown to confer insecticide resistance [119–122], and conversely downregulation occurs in response to pro-insecticides [123, 124].

A total of 58 full-length P450 genes were identified in the *O. laevigatus* genome, 11 P450 fragment genes were also found as well as 1 pseudogene. (Sequences are included in Additional file 4). These sequences were named by Dr. David Nelson using his in-house pipeline [84]. The majority of these genes (34) belonged to the diverse CYP3 class, which was a similar size to other hemipteran species (Table 10).

**Table 10:** Total numbers of Cytochrome P450 genes annotated in *Orius laevigatus* (this study), *Cimex lectularius* [86], *Rhodnius prolixus, Triatoma infestans* [96], *Frankliniella occidentalis, Thrips palmi* [90], *Myzus persicae, Acyrthosiphon pisum* [95], *Trialeurodes vaporariorum* [93], *Bemisia tabaci* [125], *Halyomorpha halys* [23] and *Murgantia histrionica* [24]

	*O. laevigatus* + close relatives				Crop pests							
	***O. laevigatus***	***C. lectularius***	***R. prolixus***	***T. infestans***	***F. occidentalis***	***T. palmi***	***M. persicae***	***A. pisum***	***T. vaporariorum***	***B. tabaci***	***H. halys***	***M. histrionica***
**CYP2**	6	6	7	1	12	12	3	10	7	18	6	7
**CYP3**	34 (41)*	36	55	65	22	26	63	33	41	76	84	43
CYP6	11	10**	8	15	18	–	–	29	34	47	–	–
CYP9	0	0**	0	0	0	–	–	0	0	0	–	–
Other	23	26**	47	50	4	–	–	4	7	–	–	–
**CYP4**	13 (17)*	11	49	22	37	42	48	32	25	73	45	30
**Mito**	5	6	8	6	10	11	1	8	7	4	6	6
**Total**	**58**	**59**	**119**	**94**	**81**	**91**	**115**	**83**	**80**	**171**	**141**	**86**

*Values in brackets represent total gene numbers including partial and fragment genes. For other species partial and fragment p450 genes were excluded in cases where they were listed as such - some may remain in the counts if official naming and curation had not taken place

**Values used are those from [86], but values differed by study [126] identified 5 CYP9s, 35 CYP6s and 5 others [127]; identified 0 CYP9s, 8 CYP6s and 15 others (these were also officially named by David Nelson)

The CYP3 clade is currently the P450 clade most associated with insecticide resistance - notably the CYP6 and CYP9 families [128]. Interestingly the CYP9 family was not present in *O. laevigatus*, as found for *T. infestans*, *R. prolixus, M. histrionica* and *H. halys* [23, 24, 96]. Further investigation into the assignment of classes within the CYP3 clade suggests the lack of the CYP9 class could be a common feature within Hemiptera (Table 10).

**Fig. 6:**
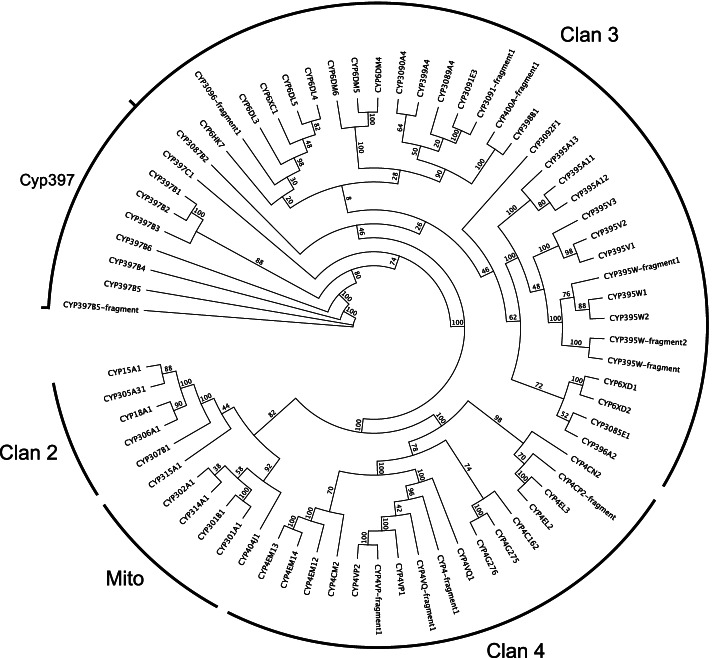
Phylogenetic tree of the *Orius laevigatus* cytochrome P450s. The Cyp397 gene family is a member of clan 3. Amino acid sequences were aligned using MAFFT and analysed using RAxML (the GAMMA LG protein model was used). The bootstrap consensus tree was inferred from 100 replicates

Expansion of the CYP397 gene family was seen in *O. laevigatus*, (Fig. 6) with 7 full-length CYP397 genes and 1 fragment. CYP397B1, CYP397B2, CYP397B6 and CYP397C1 were directly adjacent on the same scaffold, indicating tandem duplications. Sequence similarity of the CYP397 genes to CYP397B1 ranged from 52 to 86%, which suggests a variation in ages of these tandem duplications. *Cimex lectularius* also showed an increased copy number of CYP397 with 6 copies (A1-A6) [86]. CYP397A1 is significantly upregulated (> 36 fold) in pyrethroid-resistant strains of *C. lectularius* [127], therefore the expansion of this gene family could potentially confer some tolerance to pyrethroids in *O. laevigatus*.

A previous study [129] looked at the effect of insecticide synergists on *Orius tristicolor* (another minute pirate bug of the Anthocoridae family), and found that PBO (an inhibitor of P450s and esterases) significantly increased the mortality rate when combined with indoxacarb (an oxadiazine insecticide). Whereas inhibition of solely GSTs or esterases did not reduce mortality. Upregulation of P450s, esterases and GSTs have all been seen in response to oxadiazines [130], therefore the fact that only P450 inhibition had an impact on mortality rate suggests P450s may be the primary detoxification mechanism of *O. laevigatus*.

#### Target site mutations

Point mutations resulting in amino acid substitutions in the target proteins of insecticides have been characterised in many insecticide resistant insect species, including in the sodium channel gene *para* which confers resistance to pyrethroids [131]; the acetylcholinesterase-1 (ace-1) enzyme associated with organophosphate resistance [132] and the acetyl-coenzyme A Carboxylase (ACC) enzyme linked to keto-enol (spirotetramat) resistance [133]. Despite these mutations having been observed in a variety of hemipteran crop pests, none were observed in this *O. laevigatus* assembly. Although, it is important to note that the *O. laevigatus* assembly was a consensus of ~ 1000 individuals, therefore differences in target sites would likely only be apparent if they were present in the majority of the population. Overall, tolerance of insecticides by *O. laevigatus* resulting from target site differences seems unlikely compared to what is seen in crop pests, where there has been intensive selection pressure.

**Fig. 7:**
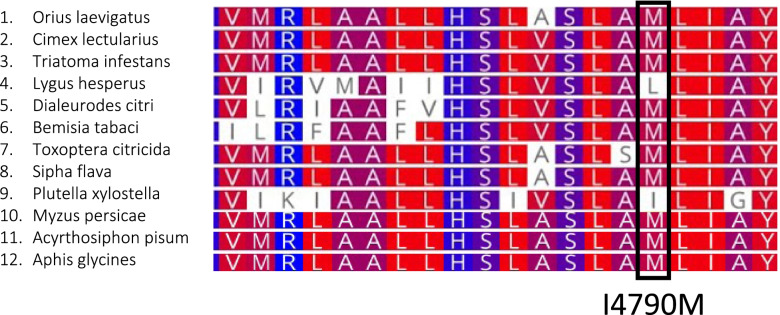
An alignment of amino acid sequences to compare transmembrane domain 3 of the conserved ryanodine receptor (RyR) from *Plutella xylostella* with hemipteran species. The box indicates the I4790M RyR point mutation linked to diamide resistance (numbered according to PxRyR). A strain of *P. xylostella* without the point mutation was used for the alignment. RyR sequences were obtained from the UniProt database, excluding *Orius laevigatus*. UniProt entry names are as follows: *Cimex lectularius*: A0A7E4RNZ4_CIMLE, *Triatoma infestans*: A0A023F678_TRIIF, *Lygus hesperus*: A0A146M8X0_LYGHE, *Dialeurodes citri*: A0A141BN13_DIACT, *Bemisia tabaci*: A0A1U9JHP1_BEMTA, *Toxoptera citricida*: A0A0H3XSN1_TOXCI, *Sipha flava:* A0A2S2QG40_9HEMI, *Plutella xylostella*: I3NWV8_PLUXY, *Myzus persicae:* A0A0A7RS32_MYZPE, *Acyrthosiphon pisum*: X1WXB1_ACYPI, *Aphis glycines:* A0A6G0U418_APHGL

The ryanodine receptor (RyR) is the target of diamide insecticides, and two target site resistance mutations conferring amino acid substitutions (I4790M and G4946E - numbering according to *Plutella xylostella*, PxRyR) have been identified in lepidopteran pests [21, 134]. Interestingly, *O. laevigatus* has the I4790M substitution (full sequence for Ryanodine in Additional file 4) which has been shown to confer varying levels of resistance to diamides. This point mutation was also present in other hemipteran species as shown in Fig. 7 (except for *Lygus hesperus* which had an I > L mutation). I4790M has been detected in lepidopteran populations across the globe and is considered to be a ‘selectivity switch’ for diamides [135]. *O. tristicolor* showed high levels of resistance to chlorantraniliprole (a diamide insecticide) with < 5% mortality [129] with the I4790M substitution being the main cause [136]. It is therefore possible that I4790M may confer some tolerance to diamides in *O. laevigatus,* and indeed, diamide resistance has been reported in *O. laevigatus* [137]. However, I4790M could potentially also confer diamide tolerance in crop pests - diamide resistance has already been shown in *F. occidentalis* [137]. Therefore this would likely not be an exploitable difference for IPM strategies.

## Conclusions

PacBio long-read technology combined with low error-rate short-read Illumina sequencing enabled the production of a high-quality genome and mitochondrial assembly for *O. laevigatus.* Whilst genome continuity may not be as good as an assembly generated from a single insect, the genome completeness is still of a sufficient quality to aid with comparative and functional genomics analyses and provides a useful first reference genome for the *Anthocoridae* family. An experimental estimate to confirm genome size and Hi-C based scaffolding would likely be the next best steps to significantly improve this genome in the future.

Comparative analyses of *O. laevigatus* with hemipteran crop pests showed evidence of possible differences in xenobiotic tolerance, including a potential increase in GST-mediated tolerance of organophosphates in *O. laevigatus*, whilst GST-mediated pyrethroid tolerance may be more prevalent in crop pests. There may also be less UGT-mediated tolerance to diamides and neonicotinoids in *O. laevigatus* compared to crop pests - although, the I4790M target site mutation may confer some degree of diamide insensitivity to *O. laevigatus*.

A recent study shows that there is significant variation in the susceptibility of *O. laevigatus* to pyrethroids when a variety of wild and commercial populations are assessed [109]. This suggests that beneficial predators such as *O. laevigatus* are certainly capable of developing insecticide resistance, but a combination of factors result in resistance developing slower than in pest species. This could be due to beneficial predators having smaller population sizes, longer life cycles, less exposure to pesticides and a lack of continuous selection pressure - beneficial predators often need to be re-released each season as populations migrate to new areas in search of food sources. These differences will have resulted in a lesser degree of selection for resistance mechanisms in *O. laevigatus* and therefore any observed differences in potential sensitivity would only be at low levels. Further comparisons looking at differences in gene expansions, expression levels and key target site mutations between resistant and susceptible strains of *O. laevigatus* would provide more concrete evidence for the findings in this study.

In conclusion, this study indicates differences in potential mechanisms of resistance between crop pests and *O. laevigatus* which could be exploited when selecting targeted insecticides. An increase in the number of pesticides which are safe for beneficial predators such as *O. laevigatus* would be of significant impact to pest management, especially at a time when the list of pesticides effective against crop pests is growing ever shorter. The findings also suggest that *O. laevigatus* has the ability to develop resistance to a variety of insecticides which could be used to our advantage through the selective breeding and selection of resistant strains of *O. laevigatus* for use in IPM strategies.

## Supplementary Information

**Additional file 1 **Numbers of proteins in the *Orius laevigatus* genome annotated by the InterPro member databases. (.docx file)

**Additional file 2 **The assembly statistics at each stage in the assembly pipeline of the *Orius laevigatus* genome. (.docx file)

**Additional file 3 **Phylogenetic tree of the *Orius laevigatus* ATP-binding cassette (ABC) transporters. (.docx file)

**Additional file 4 **Protein sequences of *Orius laevigatus* insecticide resistance genes. (.xlsx file)

**Additional file 5 **Phylogenetic tree of *Orius laevigatus, Rhodnius prolixus and Acyrthosiphon pisum* Carboxyl/cholinesterases (CCEs). (.docx file)

## Data Availability

The genome assembly generated in this study, as well as the raw PacBio and Illumina data used, are available under the BioProject accessions: PRJNA721944 and PRJEB38143. The curated *Orius laevigatus* genes used for comparative analyses are included in the additional files.

## References

[1] Oerke E-C (2006). Crop losses to pests. J Agric Sci.

[2] Geiger F, Bengtsson J, Berendse F, Weisser WW, Emmerson M, Morales MB (2010). Persistent negative effects of pesticides on biodiversity and biological control potential on European farmland. Basic Appl Ecol.

[3] Bottrell DG, Schoenly KG (2012). Resurrecting the ghost of green revolutions past: the brown planthopper as a recurring threat to high-yielding rice production in tropical Asia. J Asia Pac Entomol.

[4] Debach P, Rosen D (1992). Biological control by natural enemies (second edition). J Trop Ecol.

[5] Hernandez LM (1999). A review of the economically important species of the genus Orius (Heteroptera: Anthocoridae) in East Africa. J Nat Hist.

[6] Kelton LA (1978). The Anthocoridae of Canada and Alaska: Heteroptera.

[7] Malais M (1992). Knowing and recognizing: the biology of glasshouse pests and their natural enemies.

[8] Chambers RJ, Long S, Helyer NL (1993). Effectiveness of Orius laevigatus(hem.: Anthocoridae) for the control of Frankliniella occidentalis on cucumber and pepper in the UK. Biocontrol Sci Tech.

[9] Sanchez JA, Sanchez JA, Lacasa A (2002). Modelling population dynamics of Orius laevigatus and O. albidipennis (Hemiptera: Anthocoridae) to optimize their use as biological control agents of Frankliniella occidentalis (Thysanoptera: Thripidae). Bull Entomol Res.

[10] Tommasini MG, Maini S, Nicoli G (1997). Advances in the integrated pest management in protected-eggplant crops by seasonal inoculative releases of Orius laevigatus. Adv Hortic Sci.

[11] Hoy MA, Waterhouse RM, Wu K, Estep AS, Ioannidis P, Palmer WJ (2016). Genome sequencing of the Phytoseiid predatory mite Metaseiulus occidentalis reveals completely atomized Hox genes and Superdynamic intron evolution. Genome Biol Evol.

[12] Werren JH, Richards S, Desjardins CA, Niehuis O, Gadau J, Colbourne JK (2010). Functional and evolutionary insights from the genomes of three parasitoid Nasonia species. Science.

[13] Ando T, Matsuda T, Goto K, Hara K, Ito A, Hirata J (2018). Repeated inversions within a pannier intron drive diversification of intraspecific colour patterns of ladybird beetles. Nat Commun.

[14] Consortium TGS, Richards S, Gibbs RA, Weinstock GM, Brown SJ, Denell R (2008). The genome of the model beetle and pest Tribolium castaneum. Nature.

[15] International Aphid Genomics Consortium (2010). Genome sequence of the pea aphid *Acyrthosiphon pisum*. PLoS Biol.

[16] You M, Yue Z, He W, Yang X, Yang G, Xie M (2013). A heterozygous moth genome provides insights into herbivory and detoxification. Nat Genet.

[17] Xue J, Zhou X, Zhang C-X, Yu L-L, Fan H-W, Wang Z (2014). Genomes of the rice pest brown planthopper and its endosymbionts reveal complex complementary contributions for host adaptation. Genome Biol.

[18] Nicholson SJ, Nickerson ML, Dean M, Song Y, Hoyt PR, Rhee H (2015). The genome of Diuraphis noxia , a global aphid pest of small grains. BMC Genomics.

[19] Chen W, Hasegawa DK, Kaur N, Kliot A, Pinheiro PV, Luan J (2016). The draft genome of whitefly Bemisia tabaci MEAM1, a global crop pest, provides novel insights into virus transmission, host adaptation, and insecticide resistance. BMC Biol.

[20] Wenger JA, Cassone BJ, Legeai F, Johnston JS, Bansal R, Yates AD (2020). Whole genome sequence of the soybean aphid. Aphis glycines Insect Biochem Mol Biol.

[21] Wang X, Cao X, Jiang D, Yang Y, Wu Y (2020). CRISPR/Cas9 mediated ryanodine receptor I4790M knockin confers unequal resistance to diamides in Plutella xylostella. Insect Biochem Mol Biol.

[22] Schoville SD, Chen YH, Andersson MN, Benoit JB, Bhandari A, Bowsher JH (2018). A model species for agricultural pest genomics: the genome of the Colorado potato beetle, Leptinotarsa decemlineata (Coleoptera: Chrysomelidae). Sci Rep.

[23] Sparks ME, Bansal R, Benoit JB, Blackburn MB, Chao H, Chen M (2020). Brown marmorated stink bug, Halyomorpha halys (Stål), genome: putative underpinnings of polyphagy, insecticide resistance potential and biology of a top worldwide pest. BMC Genomics.

[24] Sparks ME, Rhoades JH, Nelson DR, Kuhar D, Lancaster J, Lehner B, et al. A Transcriptome survey spanning life stages and sexes of the harlequin bug, Murgantia histrionica. Insects. 2017;8. 10.3390/insects8020055. 10.3390/insects8020055 PMC5492069 28587099

[25] Cao C, Sun L, Wen R, Shang Q, Ma L, Wang Z (2015). Characterization of the transcriptome of the Asian gypsy moth Lymantria dispar identifies numerous transcripts associated with insecticide resistance. Pestic Biochem Physiol.

[26] Sparks ME, Nelson DR, Haber AI, Weber DC, Harrison RL (2020). Transcriptome Sequencing of the Striped Cucumber Beetle, *Acalymma vittatum* (F.), Reveals Numerous Sex-Specific Transcripts and Xenobiotic Detoxification Genes. BioTech.

[27] European Commission (2009). Directive 2009/128/EC on the sustainable use of pesticides.

[28] Cameron PJ, Walker GP, Hodson AJ, Kale AJ, Herman TJB (2009). Trends in IPM and insecticide use in processing tomatoes in New Zealand. Crop Prot.

[29] Kranthi KR, Russell DA, Peshin R, Dhawan AK (2009). Changing trends in cotton Pest management. Integrated Pest management: innovation-development process: volume 1.

[30] Meissle M, Mouron P, Musa T, Bigler F, Pons X, Vasileiadis VP (2009). Pests, pesticide use and alternative options in European maize production: current status and future prospects. J Appl Entomol.

[31] Hillocks RJ (2012). Farming with fewer pesticides: EU pesticide review and resulting challenges for UK agriculture. Crop Prot.

[32] Lechenet M, Dessaint F, Py G, Makowski D, Munier-Jolain N (2017). Reducing pesticide use while preserving crop productivity and profitability on arable farms. Nature Plants.

[33] Lattin JD, BIONOMICS OF, THE ANTHOCORIDAE (1999). Annu Rev Entomol.

[34] Heckel DG (2012). Insecticide resistance after silent spring. Science.

[35] Li X, Shi H, Gao X, Liang P (2018). Characterization of UDP-glucuronosyltransferase genes and their possible roles in multi-insecticide resistance in Plutella xylostella (L.). Pest Manag Sci.

[36] Merzendorfer H. Chapter One - ABC Transporters and Their Role in Protecting Insects from Pesticides and Their Metabolites. In: Cohen E, editor. Advances in Insect Physiology, vol. 46, Academic Press; 2014, p. 1–72.

[37] Pavlidi N, Vontas J, Van Leeuwen T (2018). The role of glutathione S-transferases (GSTs) in insecticide resistance in crop pests and disease vectors. Curr Opin Insect Sci.

[38] Scott JG (1999). Cytochromes P450 and insecticide resistance. Insect Biochem Mol Biol.

[39] Sogorb MA, Vilanova E (2002). Enzymes involved in the detoxification of organophosphorus, carbamate and pyrethroid insecticides through hydrolysis. Toxicol Lett.

[40] Rane RV, Ghodke AB, Hoffmann AA, Edwards OR, Walsh TK, Oakeshott JG (2019). Detoxifying enzyme complements and host use phenotypes in 160 insect species. Curr Opin Insect Science.

[41] Roderick GK, Navajas M (2003). Genes in new environments: genetics and evolution in biological control. Nat Rev Genet.

[42] Marçais G, Kingsford C (2011). A fast, lock-free approach for efficient parallel counting of occurrences of k-mers. Bioinformatics.

[43] Rhyker Ranallo-Benavidez T, Jaron KS, Schatz MC (2020). GenomeScope 2.0 and Smudgeplot for reference-free profiling of polyploid genomes. Nat Commun.

[44] Andrews S. FastQC n.d. https://github.com/s-andrews/FastQC (accessed 20 Apr 2021).

[45] Bolger AM, Lohse M, Usadel B (2014). Trimmomatic: a flexible trimmer for Illumina sequence data. Bioinformatics.

[46] Bradnam K (2011). A script to calculate a basic set of metrics from a genome assembly.

[47] Simão FA, Waterhouse RM, Ioannidis P, Kriventseva EV, Zdobnov EM (2015). BUSCO: assessing genome assembly and annotation completeness with single-copy orthologs. Bioinformatics.

[48] Kolmogorov M, Yuan J, Lin Y, Pevzner PA (2019). Assembly of long, error-prone reads using repeat graphs. Nat Biotechnol.

[49] Lin Y, Yuan J, Kolmogorov M, Shen MW, Chaisson M, Pevzner PA (2016). Assembly of long error-prone reads using de Bruijn graphs. Proc Natl Acad Sci U S A.

[50] Song L, Shankar DS, Florea L. Rascaf: improving genome assembly with RNA sequencing data. Plant Genome. 2016;9. 10.3835/plantgenome2016.03.0027. 10.3835/plantgenome2016.03.0027 27902792

[51] Koren S, Walenz BP, Berlin K, Miller JR, Bergman NH, Phillippy AM (2017). Canu: scalable and accurate long-read assembly via adaptive k-mer weighting and repeat separation. Genome Res.

[52] Chin C-S, Alexander DH, Marks P, Klammer AA, Drake J, Heiner C (2013). Nonhybrid, finished microbial genome assemblies from long-read SMRT sequencing data. Nat Methods.

[53] Chin C-S, Peluso P, Sedlazeck FJ, Nattestad M, Concepcion GT, Clum A (2016). Phased diploid genome assembly with single-molecule real-time sequencing. Nat Methods.

[54] Chakraborty M, Baldwin-Brown JG, Long AD, Emerson JJ (2016). Contiguous and accurate de novo assembly of metazoan genomes with modest long read coverage. Nucleic Acids Res.

[55] Walker BJ, Abeel T, Shea T, Priest M, Abouelliel A, Sakthikumar S (2014). Pilon: an integrated tool for comprehensive microbial variant detection and genome assembly improvement. PLoS One.

[56] Pryszcz LP, Gabaldón T (2016). Redundans: an assembly pipeline for highly heterozygous genomes. Nucleic Acids Res.

[57] Marçais G, Delcher AL, Phillippy AM, Coston R, Salzberg SL, Zimin A (2018). MUMmer4: A fast and versatile genome alignment system. PLoS Comput Biol.

[58] O’Leary NA, Wright MW, Brister JR, Ciufo S, Haddad D, McVeigh R (2016). Reference sequence (RefSeq) database at NCBI: current status, taxonomic expansion, and functional annotation. Nucleic Acids Res.

[59] Huson DH, Auch AF, Qi J, Schuster SC (2007). MEGAN analysis of metagenomic data. Genome Res.

[60] Du B-Z, Niu F-F, Wei S-J (2016). The complete mitochondrial genome of the predatory bug Orius sauteri (Poppius) (Hemiptera: Anthocoridae). Mitochondrial DNA Part A.

[61] Holt C, Yandell M (2011). MAKER2: an annotation pipeline and genome-database management tool for second-generation genome projects. BMC Bioinformatics.

[62] Stanke M, Steinkamp R, Waack S, Morgenstern B (2004). AUGUSTUS: a web server for gene finding in eukaryotes. Nucleic Acids Res.

[63] Lomsadze A, Ter-Hovhannisyan V, Chernoff YO, Borodovsky M (2005). Gene identification in novel eukaryotic genomes by self-training algorithm. Nucleic Acids Res.

[64] Solovyev V, Balding DJ, Bishop M, Cannings C (2001). Statistical approaches in eukaryotic gene prediction. Handbook of statistical genetics.

[65] Haas BJ, Salzberg SL, Zhu W, Pertea M, Allen JE, Orvis J (2008). Automated eukaryotic gene structure annotation using EVidenceModeler and the program to assemble spliced alignments. Genome Biol.

[66] Smit AFA, Hubley R. RepeatModeler Open-1.0 2008–2015. http://www.repeatmasker.org.

[67] Wheeler TJ, Eddy SR (2013). Nhmmer: DNA homology search with profile HMMs. Bioinformatics.

[68] Finn RD, Bateman A, Clements J, Coggill P, Eberhardt RY, Eddy SR (2014). Pfam: the protein families database. Nucleic Acids Res.

[69] Smit AFA, Hubley R, Green P. RepeatMasker Open-4.0 2013–2015. http://www.repeatmasker.org.

[70] Kim D, Langmead B, Salzberg SL (2015). HISAT: a fast spliced aligner with low memory requirements. Nat Methods.

[71] Pertea M, Pertea GM, Antonescu CM, Chang T-C, Mendell JT, Salzberg SL (2015). StringTie enables improved reconstruction of a transcriptome from RNA-seq reads. Nat Biotechnol.

[72] Grabherr MG, Haas BJ, Yassour M, Levin JZ, Thompson DA, Amit I (2011). Full-length transcriptome assembly from RNA-Seq data without a reference genome. Nat Biotechnol.

[73] Gilbert D (2013). EvidentialGene - evidence directed gene construction for eukaryotes.

[74] Götz S, García-Gómez JM, Terol J, Williams TD, Nagaraj SH, Nueda MJ (2008). High-throughput functional annotation and data mining with the Blast2GO suite. Nucleic Acids Res.

[75] Nawrocki EP, Eddy SR (2013). Infernal 1.1: 100-fold faster RNA homology searches. Bioinformatics.

[76] Bernt M, Donath A, Jühling F, Externbrink F, Florentz C, Fritzsch G (2013). MITOS: Improved de novo metazoan mitochondrial genome annotation. Mol Phylogenet Evol.

[77] Emms DM, Kelly S (2019). OrthoFinder: phylogenetic orthology inference for comparative genomics. Genome Biol.

[78] Emms DM, Kelly S. STAG: Species Tree Inference from All Genes. bioRxiv 2018:267914. 10.1101/267914.

[79] Katoh K, Standley DM (2013). MAFFT multiple sequence alignment software version 7: improvements in performance and usability. Mol Biol Evol.

[80] Katoh K, Misawa K, Kuma K-I, Miyata T (2002). MAFFT: a novel method for rapid multiple sequence alignment based on fast Fourier transform. Nucleic Acids Res.

[81] Stamatakis A (2014). RAxML version 8: a tool for phylogenetic analysis and post-analysis of large phylogenies. Bioinformatics.

[82] Le SQ, Gascuel O (2008). An improved general amino acid replacement matrix. Mol Biol Evol.

[83] Robinson JT, Thorvaldsdóttir H, Winckler W, Guttman M, Lander ES, Getz G (2011). Integrative genomics viewer. Nat Biotechnol.

[84] Nelson DR (2009). The cytochrome P450 homepage. Hum Genomics.

[85] UGT Committee. UGT Committee Home UGT Committee home n.d. https://prime.vetmed.wsu.edu/resources/udp-glucuronsyltransferase-homepage (accessed 25 Mar 2021).

[86] Rosenfeld JA, Reeves D, Brugler MR, Narechania A, Simon S, Durrett R (2016). Genome assembly and geospatial phylogenomics of the bed bug Cimex lectularius. Nat Commun.

[87] Mesquita RD, Vionette-Amaral RJ, Lowenberger C, Rivera-Pomar R, Monteiro FA, Minx P (2015). Genome of Rhodnius prolixus, an insect vector of Chagas disease, reveals unique adaptations to hematophagy and parasite infection. Proc Natl Acad Sci U S A.

[88] Dermauw W, Van Leeuwen T (2014). The ABC gene family in arthropods: comparative genomics and role in insecticide transport and resistance. Insect Biochem Mol Biol.

[89] Joe Hull J, Chaney K, Geib SM, Fabrick JA, Brent CS, Walsh D (2014). Transcriptome-based identification of ABC transporters in the Western tarnished plant bug Lygus hesperus. PLoS One.

[90] Rotenberg D, Baumann AA, Ben-Mahmoud S, Christiaens O, Dermauw W, Ioannidis P (2020). Genome-enabled insights into the biology of thrips as crop pests. BMC Biol.

[91] Guo S-K, Cao L-J, Song W, Shi P, Gao Y-F, Gong Y-J (2020). Chromosome-level assembly of the melon thrips genome yields insights into evolution of a sap-sucking lifestyle and pesticide resistance. Mol Ecol Resour.

[92] Pan Y, Zeng X, Wen S, Gao X, Liu X, Tian F (2020). Multiple ATP-binding cassette transporters genes are involved in thiamethoxam resistance in Aphis gossypii glover. Pestic Biochem Physiol.

[93] Pym A, Singh KS, Nordgren Å, Emyr Davies TG, Zimmer CT, Elias J (2019). Host plant adaptation in the polyphagous whitefly, *Trialeurodes vaporariorum* , is associated with transcriptional plasticity and altered sensitivity to insecticides. BMC Genomics.

[94] Tian L, Song T, He R, Zeng Y, Xie W, Wu Q (2017). Genome-wide analysis of ATP-binding cassette (ABC) transporters in the sweetpotato whitefly, Bemisia tabaci. BMC Genomics.

[95] Ramsey JS, Rider DS, Walsh TK, De Vos M, Gordon KHJ, Ponnala L (2010). Comparative analysis of detoxification enzymes in Acyrthosiphon pisum and Myzus persicae. Insect Mol Biol.

[96] Traverso L, Lavore A, Sierra I, Palacio V, Martinez-Barnetche J, Latorre-Estivalis JM (2017). Comparative and functional triatomine genomics reveals reductions and expansions in insecticide resistance-related gene families. PLoS Negl Trop Dis.

[97] Gawande ND, Subashini S, Murugan M, Subbarayalu M (2014). Molecular screening of insecticides with sigma glutathione S-transferases (GST) in cotton aphid Aphis gossypii using docking. Bioinformation.

[98] Lumjuan N, Rajatileka S, Changsom D, Wicheer J, Leelapat P, Prapanthadara L-A (2011). The role of the Aedes aegypti Epsilon glutathione transferases in conferring resistance to DDT and pyrethroid insecticides. Insect Biochem Mol Biol.

[99] Vontas JG, Small GJ, Hemingway J (2001). Glutathione S-transferases as antioxidant defence agents confer pyrethroid resistance in Nilaparvata lugens. Biochem J.

[100] Friedman R (2011). Genomic organization of the glutathione S-transferase family in insects. Mol Phylogenet Evol.

[101] Adelman ZN, Kilcullen KA, Koganemaru R, Anderson MAE, Anderson TD, Miller DM (2011). Deep sequencing of pyrethroid-resistant bed bugs reveals multiple mechanisms of resistance within a single population. PLoS One.

[102] Aidlin Harari O, Santos-Garcia D, Musseri M, Moshitzky P, Patel M, Visendi P (2020). Molecular evolution of the glutathione S-Transferase family in the Bemisia tabaci species complex. Genome Biol Evol.

[103] Schama R, Pedrini N, Juárez MP, Nelson DR, Torres AQ, Valle D (2016). Rhodnius prolixus supergene families of enzymes potentially associated with insecticide resistance. Insect Biochem Mol Biol.

[104] Xia J, Xu H, Yang Z, Pan H, Yang X, Guo Z, et al. Genome-wide analysis of Carboxylesterases (COEs) in the whitefly, (Gennadius). Int J Mol Sci. 2019;20 10.3390/ijms20204973. 10.3390/ijms20204973 PMC6829539 31600879

[105] Karatolos N. Molecular mechanisms of insecticide resistance in the greenhouse whitefly, *Trialeurodes vaporariorum*. PhD. University of Exeter, 2011. https://doi.org/https://ore.exeter.ac.uk/repository/bitstream/handle/10036/3350/KaratolosN.pdf.

[106] Oakeshott J, Claudianos C, Campbell PM, Newcomb RD, Russell RJ, Gilbert LI, Gill SS, Iatrou K (2010). Biochemical genetics and genomics of insect esterases. Insect pharmacology: channels, Receptors, Toxins and Enzymes, Elsevier.

[107] Flores AE, Albeldaño-Vázquez W, Salas IF, Badii MH, Becerra HL, Garcia GP (2005). Elevated α-esterase levels associated with permethrin tolerance in Aedes aegypti (L.) from Baja California, Mexico. Pestic Biochem Physiol.

[108] Orihuela PLS, Vassena CV, Zerba EN, Picollo MI (2014). Relative contribution of Monooxygenase and esterase to Pyrethroid resistance in Triatoma infestans (Hemiptera: Reduviidae) from Argentina and Bolivia. J Med Entomol.

[109] Balanza V, Mendoza JE, Cifuentes D, Bielza P (2021). Selection for resistance to pyrethroids in the predator Orius laevigatus. Pest Manag Sci.

[110] Ganesh KN, Vijayan VA, Urmila J, Gopalan N, Prakash S (2002). Role of esterases and monooxygenase in the deltamethrin resistance in Anopheles stephensi Giles (1908), at Mysore. Indian J Exp Biol.

[111] Prasad KM, Raghavendra K, Verma V, Velamuri PS, Pande V (2017). Esterases are responsible for malathion resistance in Anopheles stephensi: a proof using biochemical and insecticide inhibition studies. J Vector Borne Dis.

[112] Jyoti SNK, Singh H, Singh NK, Rath SS (2016). Multiple mutations in the acetylcholinesterase 3 gene associated with organophosphate resistance in Rhipicephalus (Boophilus) microplus ticks from Punjab, India. Vet Parasitol.

[113] Zhang Y, Li S, Xu L, Guo HF, Zi J, Wang L (2013). Overexpression of carboxylesterase-1 and mutation (F439H) of acetylcholinesterase-1 are associated with chlorpyrifos resistance in Laodelphax striatellus. Pestic Biochem Physiol.

[114] Zhao J, Xu L, Sun Y, Song P, Han Z. UDP-Glycosyltransferase genes in the striped Rice stem borer, (Walker), and their contribution to Chlorantraniliprole resistance. Int J Mol Sci. 2019;20 10.3390/ijms20051064. 10.3390/ijms20051064 PMC6429375 30823656

[115] Tian F, Wang Z, Li C, Liu J, Zeng X (2019). UDP-Glycosyltransferases are involved in imidacloprid resistance in the Asian citrus psyllid, Diaphorina citri (Hemiptera: Lividae). Pestic Biochem Physiol.

[116] Cui X, Wang C, Wang X, Li G, Liu Z, Wang H (2020). Molecular mechanism of the UDP-Glucuronosyltransferase 2B20-like gene (AccUGT2B20-like) in pesticide resistance of Apis cerana cerana. Front Genet.

[117] Pan Y, Xu P, Zeng X, Liu X, Shang Q (2019). Characterization of UDP-Glucuronosyltransferases and the potential contribution to nicotine tolerance in Myzus persicae. Int J Mol Sci.

[118] Xie W, He C, Fei Z, Zhang Y (2020). Chromosome-level genome assembly of the greenhouse whitefly (Trialeurodes vaporariorum Westwood). Mol Ecol Resour.

[119] Karunker I, Benting J, Lueke B, Ponge T, Nauen R, Roditakis E (2008). Over-expression of cytochrome P450 CYP6CM1 is associated with high resistance to imidacloprid in the B and Q biotypes of Bemisia tabaci (Hemiptera: Aleyrodidae). Insect Biochem Mol Biol.

[120] Liang X, Xiao D, He Y, Yao J, Zhu G, Zhu KY (2015). Insecticide-mediated up-regulation of cytochrome P450 genes in the red flour beetle (Tribolium castaneum). Int J Mol Sci.

[121] Puinean AM, Foster SP, Oliphant L, Denholm I, Field LM, Millar NS (2010). Amplification of a cytochrome P450 gene is associated with resistance to neonicotinoid insecticides in the aphid Myzus persicae. PLoS Genet.

[122] Yang T, Liu N (2011). Genome analysis of cytochrome P450s and their expression profiles in insecticide resistant mosquitoes, *Culex quinquefasciatus*. PLoS One.

[123] Main BJ, Everitt A, Cornel AJ, Hormozdiari F, Lanzaro GC (2018). Genetic variation associated with increased insecticide resistance in the malaria mosquito, Anopheles coluzzii. Parasit Vectors.

[124] Vlogiannitis S, Mavridis K, Dermauw W, Snoeck S, Katsavou E, Morou E, et al. Reduced proinsecticide activation by cytochrome P450 confers coumaphos resistance in the major bee parasite Varroa destructor. Proc Natl Acad Sci U S A. 2021;118. 10.1073/pnas.2020380118. 10.1073/pnas.2020380118 PMC8017976 33547243

[125] Ilias A, Lagnel J, Kapantaidaki DE, Roditakis E, Tsigenopoulos CS, Vontas J (2015). Transcription analysis of neonicotinoid resistance in Mediterranean (MED) populations of B. tabaci reveal novel cytochrome P450s, but no nAChR mutations associated with the phenotype. BMC Genomics.

[126] Bai X, Mamidala P, Rajarapu SP, Jones SC, Mittapalli O (2011). Transcriptomics of the bed bug (Cimex lectularius). PLoS One.

[127] Zhu F, Gujar H, Gordon JR, Haynes KF, Potter MF, Palli SR (2013). Bed bugs evolved unique adaptive strategy to resist pyrethroid insecticides. Sci Rep.

[128] Feyereisen R (2006). Evolution of insect P450. Biochem Soc Trans.

[129] Pereira RR, Picanço MC, Santana PA, Moreira SS, Guedes RNC, Corrêa AS (2014). Insecticide toxicity and walking response of three pirate bug predators of the tomato leaf minerTuta absoluta. Agric For Entomol.

[130] Shi L, Shi Y, Zhang Y, Liao X (2019). A systemic study of indoxacarb resistance in Spodoptera litura revealed complex expression profiles and regulatory mechanism. Sci Rep.

[131] Martinez-Torres D, Foster SP, Field LM, Devonshire AL, Williamson MS (1999). A sodium channel point mutation is associated with resistance to DDT and pyrethroid insecticides in the peach-potato aphid, Myzus persicae (Sulzer) (Hemiptera: Aphididae). Insect Mol Biol.

[132] Alon M, Alon F, Nauen R, Morin S (2008). Organophosphates’ resistance in the B-biotype of Bemisia tabaci (Hemiptera: Aleyrodidae) is associated with a point mutation in an ace1-type acetylcholinesterase and overexpression of carboxylesterase. Insect Biochem Mol Biol.

[133] Lueke B, Douris V, Hopkinson JE, Maiwald F, Hertlein G, Papapostolou K-M (2020). Identification and functional characterization of a novel acetyl-CoA carboxylase mutation associated with ketoenol resistance in Bemisia tabaci. Pestic Biochem Physiol.

[134] Zuo Y-Y, Ma H-H, Lu W-J, Wang X-L, Wu S-W, Nauen R (2020). Identification of the ryanodine receptor mutation I4743M and its contribution to diamide insecticide resistance in Spodoptera exigua (Lepidoptera: Noctuidae). Insect Sci.

[135] Richardson EB, Troczka BJ, Gutbrod O, Davies TGE, Nauen R (2020). Diamide resistance: 10 years of lessons from lepidopteran pests. J Pest Sci.

[136] Nauen R, Steinbach D, Horowitz AR, Ishaaya I (2016). Resistance to Diamide insecticides in Lepidopteran pests. Advances in insect control and resistance management.

[137] Dáder B, Colomer I, Adán Á, Medina P, Viñuela E (2020). Compatibility of early natural enemy introductions in commercial pepper and tomato greenhouses with repeated pesticide applications. Insect Sci.

