# Supplementary material for: A scaffold-level genome assembly of a minute pirate bug, Orius laevigatus (Hemiptera: Anthocoridae), and a comparative analysis of insecticide resistance-related gene families with hemipteran crop pests
Source: BMC Genomics. 2022 Jan 11;23:45. doi: 10.1186/s12864-021-08249-y (PMC8751118; doi:10.1186/s12864-021-08249-y)
Supplement: Supplementary file 1 — Additional file 1 Numbers of proteins in the Orius laevigatus genome annotated by the InterPro member databases. (.docx file) [file 12864_2021_8249_MOESM1_ESM.docx]

**Additional file 1. The numbers of proteins in the *Orius laevigatus* genome annotated by the InterPro member databases**

| **InterPro member database** | **Number of proteins annotated** |
| --- | --- |
| Pfam | 9,799 |
| PANTHER | 10,433 |
| CATH-GENE3D | 8,278 |
| SUPERFAMILY | 8,097 |
