# Supplementary material for: A scaffold-level genome assembly of a minute pirate bug, Orius laevigatus (Hemiptera: Anthocoridae), and a comparative analysis of insecticide resistance-related gene families with hemipteran crop pests
Source: BMC Genomics. 2022 Jan 11;23:45. doi: 10.1186/s12864-021-08249-y (PMC8751118; doi:10.1186/s12864-021-08249-y)
Supplement: Supplementary file 2 — Additional file 2 The assembly statistics at each stage in the assembly pipeline of the Orius laevigatus genome. (.docx file) [file 12864_2021_8249_MOESM2_ESM.docx]

**Additional file 2. Assembly statistics at each stage in the assembly pipeline of the *Orius laevigatus* genome**

|  | **Scaffold N50** | **Longest Scaffold** | **Number of Scaffolds** | **BUSCO Score**  **C = complete, S = single copy, D = duplicated, F = fragmented, M = missing** |
| --- | --- | --- | --- | --- |
| **Flye** | 89,878 | 1,464,728 | 2,800 | C:86.1%[S:83.8%,D:2.3%],F:4.5%,M:9.4% |
| **Flye w/ Rascaf** | 105,409 | 1,464,728 | 2,611 | C:86.1%[S:83.7%,D:2.4%],F:4.6%,M:9.3% |
| **Falcon** | 22,424 | 1,701,193 | 7,514 | C:54.3%[S:49.2%,D:5.1%],F:10.4%,M:35.3% |
| **Falcon w/ Unzip** | 43,913 | 1,667,874 | 1,665 | C:45.5%[S:43.4%,D:2.1%],F:4.3%,M:50.2% |
| **Canu** | 27,715 | 859,161 | 8,818 | C:75.8%[S:65.4%,D:10.4%],F:7.2%,M:17.0% |
| **Quickmerge: Falcon w/ Unzip + Flye w/ Rascaf** | 112,029 | 1,935,382 | 2,473 | C:85.6%[S:82.8%,D:2.8%],F:4.8%,M:9.6% |
| **Quickmerge: Falcon w/ Unzip + Flye w/ Rascaf + Canu** | 120,811 | 2,051,687 | 2,353 | C:84.5%[S:81.7%,D:2.8%],F:4.9%,M:10.6% |
| **1^st^ Round Pilon** | 121,050 | 2,051,719 | 2,353 | C:89.8%[S:86.2%,D:3.6%],F:1.9%,M:8.3% |
| **Redundans redundancy removal** | 128,051 | 2,051,719 | 2,018 | C:88.9%[S:86.8%,D:2.1%],F:2.1%,M:9.0% |
| **2^nd^ Round Pilon** | 128,042 | 2,051,825 | 2,018 | C:88.9%[S:86.7%,D:2.2%],F:2.1%,M:9.0% |
| **Redundans scaffolding** | 128,042 | 2,051,825 | 2,012 | C:88.9%[S:86.7%,D:2.2%],F:2.1%,M:9.0% |
| **3^rd^ Round Pilon** | 128,043 | 2,051,691 | 2,012 | C:89.0%[S:86.8%,D:2.2%],F:2.1%,M:8.9% |
| **Manually bringing in genes from the original Flye assembly** | 125,152 | 2,051,691 | 2,022 | C:90.9%[S:88.7%,D:2.2%],F:1.9%,M:7.2% |
| **Manually bringing in genes from the original Falcon Assembly** | 125,152 | 2,051,691 | 2,023 | C:91.1%[S:88.9%,D:2.2%],F:1.7%,M:7.2% |
| **Manually bringing in genes from the original Canu Assembly** | 125,657 | 2,051,691 | 2,050 | C:93.1%[S:91.0%,D:2.1%],F:2.0%,M:4.9% |
| **4^th^ Round Pilon (5^th^ showed no improvement)** | 125,649 | 2,051,674 | 2,050 | C:93.6%[S:91.3%,D:2.3%],F:1.6%,M:4.8% |
