# Supplementary material for: A scaffold-level genome assembly of a minute pirate bug, Orius laevigatus (Hemiptera: Anthocoridae), and a comparative analysis of insecticide resistance-related gene families with hemipteran crop pests
Source: BMC Genomics. 2022 Jan 11;23:45. doi: 10.1186/s12864-021-08249-y (PMC8751118; doi:10.1186/s12864-021-08249-y)

**Additional file 3. Phylogenetic tree of the *Orius laevigatus* ATP-binding cassette (ABC) transporters. Amino acid sequences were aligned using MAFFT and analyzed using RAxML (the GAMMA LG protein model was used). The bootstrap consensus tree was inferred from 100 replicates.**


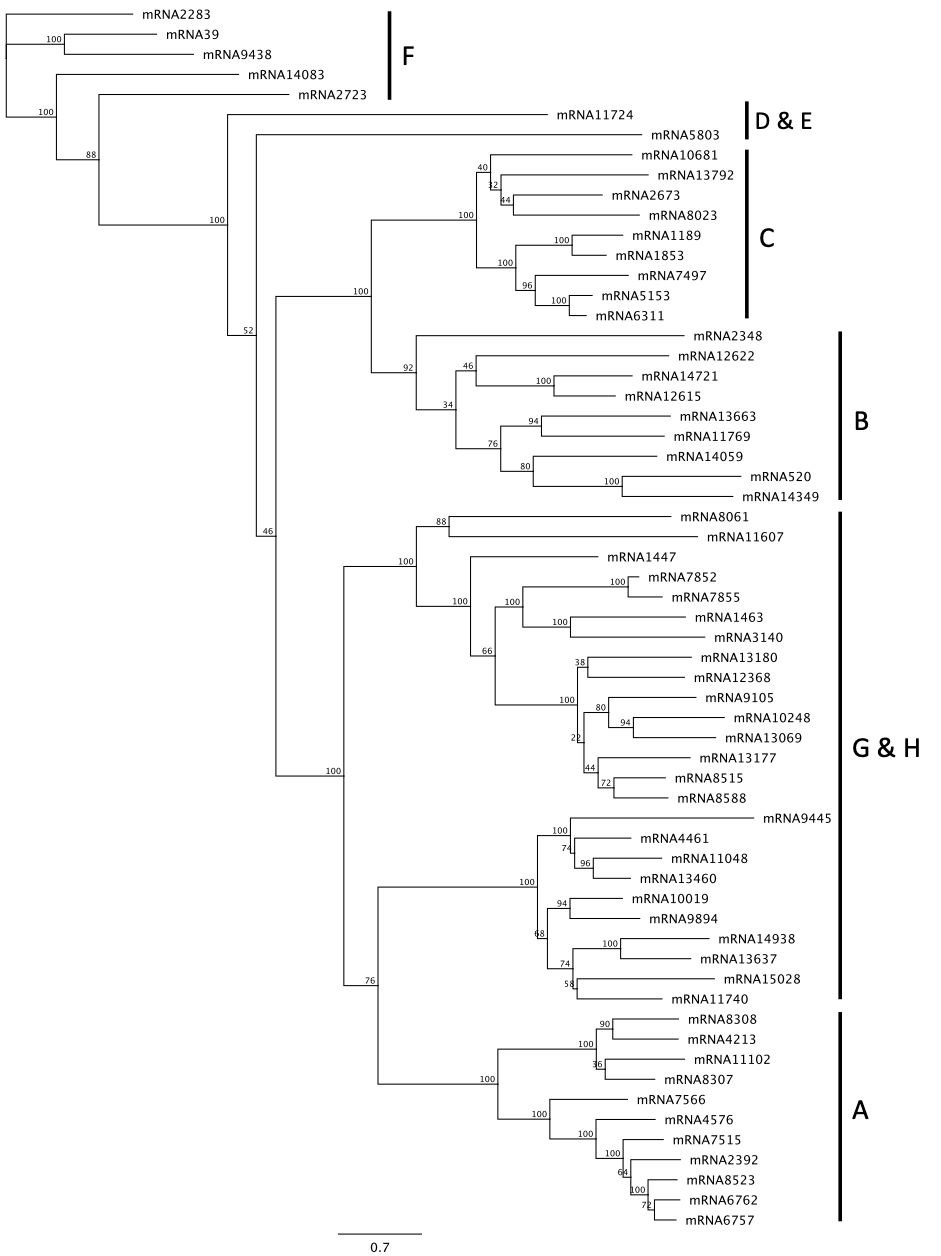

Supplement: Supplementary file 3 — Additional file 3 Phylogenetic tree of the Orius laevigatus ATP-binding cassette (ABC) transporters. (.docx file) [file 12864_2021_8249_MOESM3_ESM.docx]
