# Supplementary material for: A scaffold-level genome assembly of a minute pirate bug, Orius laevigatus (Hemiptera: Anthocoridae), and a comparative analysis of insecticide resistance-related gene families with hemipteran crop pests
Source: BMC Genomics. 2022 Jan 11;23:45. doi: 10.1186/s12864-021-08249-y (PMC8751118; doi:10.1186/s12864-021-08249-y)
Supplement: Supplementary file 5 — Additional file 5 Phylogenetic tree of Orius laevigatus, Rhodnius prolixus and Acyrthosiphon pisum Carboxyl/cholinesterases (CCEs). (.docx file) [file 12864_2021_8249_MOESM5_ESM.docx]

**Additional file 5. Phylogenetic tree of *Orius laevigatus, Rhodnius prolixus and Acyrthosiphon pisum* Carboxyl/cholinesterases (CCEs).** Amino acid sequences were aligned using MAFFT and analysed using RAxML (the GAMMA LG protein model was used). The bootstrap consensus tree was inferred from 100 replicates. *O. laevigatus* CCEs are those beginning “mRNA…”. *R. prolixus (RPRC)* and *A. pisum (ACYP)* CCEs were obtained from UniProt.
